# Tumor Budding as an Independent Prognostic Histopathological Marker in Oral Squamous Cell Carcinoma - An Indian Tertiary Care Center Experience

**DOI:** 10.5146/tjpath.2025.13761

**Published:** 2025-05-31

**Authors:** Anand Vijaya Narayanan, Krishnapriya Umashankar, Sithara Aravind, Sangeetha. K. Nayanar, Sandeep Vijay

**Affiliations:** Oncopathology Division, Department of Clinical Laboratory Services & Translational Research, Kerala, India; Department of Surgical Oncology, Malabar Cancer Centre (Post Graduate Institute of Oncology Sciences and Research), Kerela, India

**Keywords:** Oral squamous cell carcinoma, Tumor budding, Worst pattern of invasion, Depth of invasion, Perineural invasion

## Abstract

***Objective: *** Oral squamous cell carcinoma is the most common head and neck malignancy reported worldwide. Tumor budding represents a histopathological feature characterized by the presence of isolated single/small clusters of cancer cells dispersed within the stroma at the invasive tumor front. Its prognostic significance has not been studied much in lip and oral squamous cell carcinomas in India.

The aim of this study was to investigate the prognostic role of tumor budding in a large single-center retrospective cohort of 333 patients with oral squamous cell carcinoma at a tertiary cancer center in North Kerala, India.

***Material and Methods:*** The primary resection slides of 333 patients with oral squamous cell carcinoma from 2018 to 2020 were retrieved from the pathology archives and were evaluated by two independent pathologists for tumor budding and other histopathological parameters. The survival data were collected from the patient files.

***Results: *** We found a significant association between tumor budding and other known histopathological prognosticators using Chi-square analysis. Univariate logistic analysis showed tumor budding, depth of invasion (>10 mm), worst pattern of invasion 5, and perineural invasion were significantly associated with locoregional recurrence/distant metastasis. Multivariate logistic regression analysis identified tumor budding as an independent prognostic marker for locoregional recurrence/distant metastasis. Univariate cox proportionality analysis showed that tumor budding, depth of invasion (>10 mm), worst pattern of invasion 5, pathological T4 stage, and perineural invasion were associated with decreased overall survival and poor disease-free survival in patients with oral squamous cell carcinoma. Multivariate cox proportionality analysis showed tumor budding as the only independent predictor for decreased overall survival and poor disease-free survival.

***Conclusion:*** Based on this study, we can conclude that tumor budding is a simple and a reliable independent prognosticator that facilitates personalized management in patients with oral squamous cell carcinoma.

## INTRODUCTION

Tumor budding (TB) is a histological feature characterized by the presence of isolated single/small clusters of cancer cells seen within the stroma at the invasive tumor front (1). TB at the invasive front implies the dissociation of the invasive neoplastic cells from the main tumor mass (2). It is suggested that the tumor cells at the invasive tumor front undergo epithelial mesenchymal transition (EMT), which aids in the progression of metastasis (2). TB is considered to be the most important and adverse prognostic indicator in various carcinomas involving the colorectum, lungs, esophagus, bladder, pancreas, breast, and endometrium (1,3,4).

Oral squamous cell carcinoma (OSCC) is one of the most common head and neck malignancies reported worldwide (5). According to Globocan 2022, the number of new lip and oral cavity malignancies was estimated to be 389,846 worldwide (5). In India, it is the second most common type of cancer to be reported (5). India has experienced a 1.1-fold increase in the total number of new cases of lip and oral cavity cancer, and the mortality rate stands at 8.7% (5).

An array of histopathological parameters such as depth of invasion (DOI), worst pattern of invasion (WPOI), perineural invasion (PNI), lymphovascular invasion (LVI), pathological ‘T stage (pT), and extranodal extension (ENE) have proven to significantly affect the survival of patients with OSCC (6–8). Notwithstanding the development of various prognostic indicators, the accuracy of survival and prognosis prediction in OSCC remains limited. Hence there is a need for identification of simple, cost effective, and reliable microscopic parameters in routine histopathological reporting, which could aid in risk stratification of the patients with OSCC. TB is a distinctly separate histological entity that could be incorporated into routine reporting, which could aid in predicting the prognosis, as well as in risk stratification of patients with OSCC.

Although TB has been studied extensively in other solid carcinomas, a certain degree of overlap is identified between TB, and a few of the histopathological parameters described in OSCC, especially WPOI 4 & 5 (9). WPOI 4 is defined as invasive islands of less than 15 cells per island or with single infiltrating keratinocytes and WPOI 5 is defined as tumor satellites (number of cells not specified), which are seen at more than 1 mm distance from the tumor (7). However, TB is defined as cell clusters of less than or equal to 5 cells seen at the invasive tumor front (10). Based on our understanding, the definition of tumor buds does not specify the distance of the tumor buds from the main tumor, unlike WPOI 5. Additionally, not every WPOI 4 displays five cells or less. Hence TB is a distinctly separate histological entity that could be incorporated in routine reporting (9). Thus, TB is a semi-quantitative and objective parameter and WPOI is an objective and qualitative parameter (9).

Current reviews on the prognostic significance of TB in OSCC suggest that high TB was significantly associated with nodal metastasis and poor prognosis (11). However, its prognostic significance has not been studied much in OSCC, especially in the South Indian population. Hence, we aimed to investigate the prognostic role of TB in a large single-center retrospective cohort of 333 patients with OSCC at a tertiary cancer center in North Kerala.

## MATERIALS and METHODS

### Study Cohort

Institutional Review Board approval was obtained. A total of 333 patients who were diagnosed as OSCC from our tertiary cancer center in Thalassery, Kerala, India were included in the study. The sites of OSCC included in our study are mobile tongue, buccal mucosa, alveolar mucosa, retromolar trigone, gingivobuccal sulcus, floor of the mouth, and mucosal lip. The following inclusion criteria were considered: primary resection done at our institute from 2018 to 2020, histopathological diagnosis of OSCC, slides of the primary resection that were available for review, and complete clinical data that were available for review. The exclusion criteria included synchronous head and neck squamous cell carcinoma, prior history of neoadjuvant chemotherapy/radiotherapy for the OSCC, and distant metastasis during the time of presentation.

### Pathological Review

Two independent head and neck pathologists (1 Consultant and 1 Fellow) conducted a detailed histopathological evaluation of all the tumor slides. At least one tumor section per centimeter of the tumor size was evaluated (i.e., if the tumor size was 5 cm, five tumor sections were assessed) and a minimum of one tumor section with maximum tumor thickness was evaluated to identify the tumor buds at the invasive front of the tumor. The cases with discrepancy in tumor budding score were reviewed by the Senior Pathologist and a final score was assigned.

**Figure 1:**
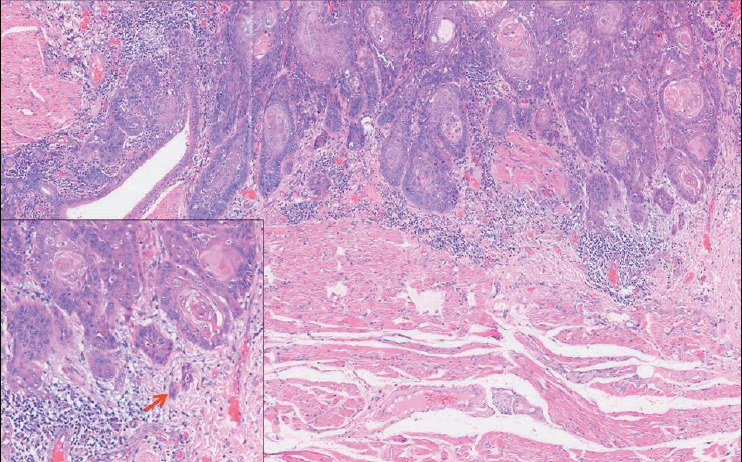
Hematoxylin & Eosin stained photomicrograph in 10x magnification shows the invasive tumor front of primary resection slide with low TB (<5 buds/x20 magnification). The inserted photomicrograph shows the 20x magnification of the invasive tumor bud with low TB. The red arrow points to the tumor buds at the invasive tumor front.

**Figure 2:**
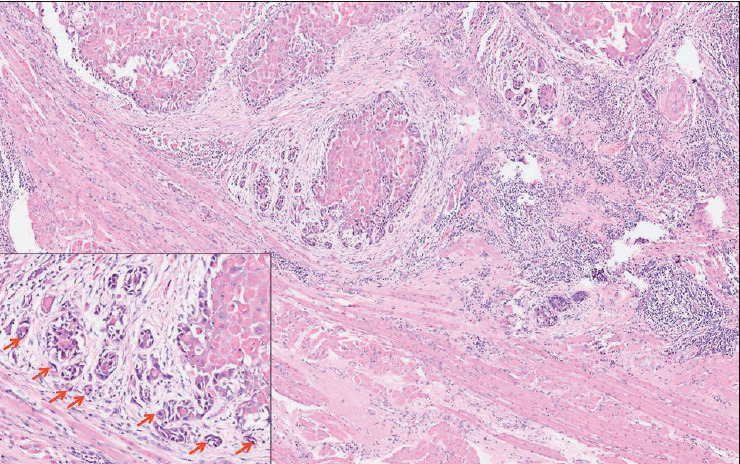
Hematoxylin & Eosin stained photomicrograph in 10x magnification shows the invasive tumor front of primary resection slide with intermediate TB (>/=5-10 buds/x20 magnification). The inserted photomicrograph shows the 20x magnification of the invasive tumor bud with intermediate TB. The red arrows point to the tumor buds at the invasive tumor front.

**Figure 3:**
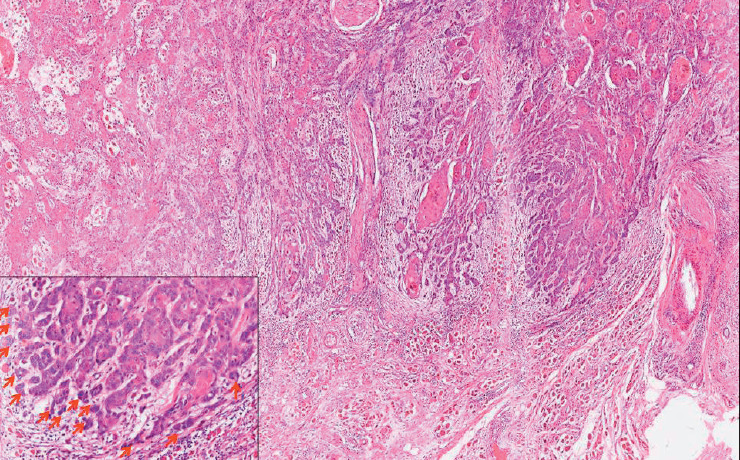
Hematoxylin & Eosin stained photomicrograph in 10x magnification shows the invasive tumor front of primary resection slide with high TB (>/=10 buds/x20 magnification). The inserted photomicrograph shows the 20x magnification of the invasive tumor bud with high TB. The red arrows point to the tumor buds at the invasive tumor front.

We evaluated TB based on the recommendations proposed by the International TB Consensus Conference (ITBCC) in 2016 for evaluating TB in colorectal carcinomas (10). The number of tumor buds (defined as a tumor cluster of < 5 tumor cells) was counted using a 20x objective with an adjusted standard field size of 0.785 mm2 at the site with the highest number of buds within the tumor (i.e., hotspot). TB was then classified as Low: 0–4 buds (Figure 1); Intermediate: 5–9 buds (Figure 2); and High: ≥10 buds (Figure 3).

DOI was measured and recorded as per the American Joint Cancer Committee (AJCC, 8th edition) recommendations and was categorized into DOI less than 5 mm, more than 5 to less than 10 mm, and greater than 10 mm (12). The pattern of invasion was evaluated based on Brandwein-Gensler recommendations (2005), Pattern 1: invasion in broad pushing manner; Pattern 2: invasion into the stroma as a broad pushing finger-like manner/large stellate tumor islands; Pattern 3: Invasive tumor islands of more than 15 cells per island; Pattern 4: Invasive tumor islands of less than 15 cells per island, irrespective of the number of the cells, and strands with single cell filing pattern; Pattern 5: Dispersed pattern with tumor satellites of any size with a distance of more than or equal to 1 mm from the main tumor mass without any fibrosis of the intervening stroma (7). Lymphocytic host response was assessed using the Brandwein-Gensler recommendations (2005); Pattern 1: Continuous and dense rim of lymphocytes at the interface, Pattern 2: Discontinuous, dense rim of lymphocytes at the interface, Pattern 3: Limited lymphocytic response or no response (7). Pathological T staging was assessed based on the AJCC 8th edition recommendations (12). PNI and LVI were recorded as either present or absent.

The lymph nodes were evaluated for metastatic deposits from squamous cell carcinoma. Extranodal extension of the involved nodes was also assessed and recorded. The survival data were collected from the physical files and electronic files of patients included in the study.

The collected data were entered into Microsoft Excel 2007 and analyzed with IBM SPSS Statistics for Windows, Version 27.0. (Armonk, NY: IBM Corp). Overall survival was calculated from the date of diagnosis to the date of death or date of final clinical follow-up. Patients who died of recurrence or distant metastasis were considered to have died because of the disease. Patients who died from medical problems during the first month of surgery were also classed as having died because of cancer. Disease Free Survival (DFS) was calculated from the time of surgery to the date of diagnosis of a locoregional recurrence/distant metastasis, or until the date of last follow-up. The Kaplan–Meier survival analysis was used to estimate survival rates. The log rank test was used to compare the survival within the groups. Univariate logistic regression was done to calculate the risk of local recurrence/distant metastasis according to TB, DOI, WPOI, T stage, PNI, LVI, and ENE. The parameters that were significant on univariate analysis were subjected to multivariate logistic regression. The Odds Ratio (OR) and 95% confidence interval (CI) were estimated. Cox proportional hazards modeling was used for both univariate and multivariate analysis to identify the effect of pathological parameters on survival outcomes. P values less than 0.05 were considered as statistically significant.

## RESULTS

### Clinicopathological characteristics of the study cohort

The predominant population in our study cohort was males (73.9%). The patients’ age ranged from 21 to 75 years (Mean: 56.9 years). The most common site of involvement was the mobile tongue (44.4%) followed by the buccal mucosa (25.5%). However, other sites involved included the alveolar mucosa (11.4%), gingivobuccal sulcus (6.3%), and floor of the mouth (5.4%), retromolar trigone (4.5%), and mucosal lip (2.4%).

We observed that high TB was seen in the mobile tongue (47.5%), followed by the buccal mucosa (26.2%), floor of the mouth (10.6%), gingivobuccal sulcus (5.7%), alveolar mucosa and palatal mucosa (3.3%), retromolar trigone (5.3%), and mucosal lip (1.4%). The chi-square test showed no significant association of high tumor budding with the sites (p=0.790).

In our study cohort, we found that 42.6% of the cases showed high TB, and 28.8% and 28.5% of the cases showed low and intermediate TB respectively. Most of the OSCC cases were graded as moderately differentiated (51.4%). WPOI 4 was seen in 52.5% of the cases and WPOI 5 was seen in 23.4% of cases. DOI greater than 10 mm was observed in 44.7% of the cases. PNI and LVI were seen in 32.7% and 1.8% cases respectively. Lymphocytic host response type II was predominant (66.1%). pT4 tumors were predominant accounting for 41.7% of the cases. Pearson chi-square test was performed to identify the association between TB and other clinicopathological parameters that included site, DOI, WPOI, LHR, PNI, LVI, pT stage, histological grade, and ENE. A significant association was identified between TB and clinicopathological parameters such as DOI, WPOI, LHR, PNI, ENE, and pT stage, using the Chi-square test (Table 1).

**Table 1:** Table depicting the clinicopathological characteristics of our study cohort

**Parameters**	**Total cases**	**Tumor budding**			**p value**
		**Low**	**Intermediate**	**High**	
**Sites**					p=0.839
Mobile tongue	148	39	42	67	
Gingivobuccal sulcus	21	5	8	8	
Alveolar mucosa	38	12	11	15	
Retromolar trigone	15	4	5	6	
Floor of the mouth	18	6	6	6	
Mucosal lip	8	5	1	2	
Buccal mucosa	85	25	22	38	
**Depth of invasion**					p<0.01*
0.1 - 5 mm	89	46	19	24	
5.1 - 10 mm	95	25	30	40	
> 10 mm	149	25	46	78	
**Worst pattern of invasion**					p<0.01*
Type 1	3	1	0	2	
Type 2	5	3	0	2	
Type 3	74	43	15	16	
Type 4	173	37	59	77	
Type 5	78	12	21	45	
**Lymphocytic host response**					p<0.01*
Type 1	106	44	36	26	
Type 2	220	51	58	111	
Type 3	7	1	1	5	
**Peri-neural invasion**					p<0.01*
Present	109	16	31	62	
Absent	224	80	64	80	
**Pathological ‘T’ stage**					p<0.01*
PT1	55	31	11	13	
pT2	81	29	27	25	
pT3	58	11	19	28	
pT4	139	25	38	76	
**Histological grade**					p>0.05
Well differentiated	147	48	40	59	
Moderately differentiated	171	44	53	74	
Poorly differentiated	15	4	2	9	
**Lymphovascular invasion**					p>0.05
Present	6	1	1	4	
Absent	327	95	94	138	
**Extranodal extension**					P<0.01*
Absent	58	9	16	33	
Present	56	5	17	34	

The p-value denotes the association of TB with the clinicopathological parameters. *p values: Significant.

### Risk of recurrence/distant metastasis according to the histopathological prognosticators in our study cohort

Seventy-seven patients developed locoregional recurrence/distant metastasis. Univariate logistic regression analysis showed that locoregional recurrence/distant metastasis was significantly associated with DOI, WPOI 5, TB, PNI but not with LVI, ENE, and TNM staging. The Odds Ratio (OR), its 95% CI and p-values are demonstrated in Table 2. Locoregional recurrence was seen to be associated only with DOI greater than 10 mm. Adjusted multivariate analysis for DOI, WPOI, TB and PNI showed that high tumor budding was significantly associated with high risk of locoregional recurrence/distant metastasis compared with low TB, (OR 12.236, 95% CI 4.129 – 36.257, p<0.05).

**Table 2:** Univariate and multivariate logistic regression model to predict the risk of locoregional recurrence/distant metastasis in our study cohort

**Parameters**	**Univariate**			**Multivariate**		
	**OR**	**95% CI**	**p value**	**OR**	**95% CI**	**p value**
**Depth of invasion**						
< 5 mm.	*Reference*			*Reference*		
5-10 mm.	1.367	0.626 - 2.983	0.433	0.895	0.387 - 2.071	0.796
> 10 mm.	2.53	1.276 - 5.015	0.008*	1.428	0.660 - 3.091	0.366
**Worst pattern of invasion**						
WPOI 1, 2, 3, 4	*Reference*			*Reference*		
WPOI 5	1.735	0.982 - 3.066	0.058*	1.169	0.628 - 2.178	0.622
**Tumor budding**						
Low	*Reference*			*Reference*		
Intermediate	5.75	1.876 - 17.626	0.002*	5.301	1.696 - 16.563	0.004*
High	13.697	4.758 - 39.425	<0.001*	12.236	4.129 - 36.257	<0.001*
**Perineural invasion**						
Absent	*Reference*			*Reference*		
Present	1.581	0.931 - 2.684	0.09*	0.97	0.540 - 1.743	0.918
**Lymphovascular invasion**						
Absent	*Reference*			*Not done*		
Present	0	-	0.999			
***TNM staging***						
PT1, T2, T3	*Reference*			*Not done*		
PT4	1.346	0.804 - 2.252	0.258			
**Extranodal extension**						
Absent	*Reference*			*Not done*		
Present	1.024	0.453 - 2.316	0.954			

*p values: Significant. **OR:** Odds ratio, **CI: **Confidence interval.

### The prognostic significance of TB and other known histopathological parameters in our study cohort

The median overall and disease free survival of our cohort was not attained (Range: 1-63 months). The estimates for the three- and five-year OS were 67.5% and 73.1%, respectively. Estimates for the 3 year and 5 year DFS were 52.2% and 73.4%, respectively. Thirty patients reported distant metastases, forty-seven patients had locoregional recurrences, and 256 patients were still alive at the time of the last follow-up. A total of 55 patients (38.7%) with high TB presented with distant metastases or locoregional recurrence.

**Figure 4:**
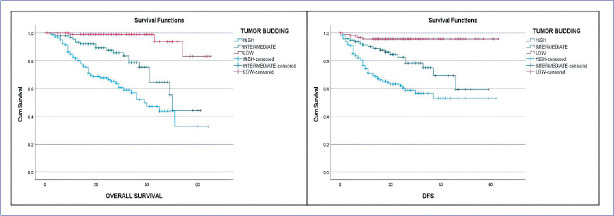
Kaplan Meier survival curves overall survival and disease free survival among OSCC patients (Censored). The curves show a significant drop in the OS and DFS in patients with high TB.

The 3 year OS for low, intermediate, and high TB cases was estimated to be 98.9%, 78.9%, and 56.9%, respectively. The 3 year DFS for low, intermediate, and high TB cases was estimated to be 95.7%, 75%, and 56.6%, respectively. Similarly, the 5 year OS for low, intermediate, and high TB cases was estimated to be 98.9%, 44.3%, and 32.8%, respectively. The 5 year DFS for low, intermediate, and high TB cases was estimated to be 95.7%, 59.4%, and 53%, respectively. Log rank analysis was performed among the different grades of TB and was found to be statistically significant (p<0.05) (Figure 4).

The results of our univariate and multivariate analysis using the Cox-proportionality hazards model is demonstrated in Table 3. DOI greater than 10 mm, WPOI 5, high TB, PNI, and pT4 tumor stage were found to be significant prognostic factors for OS (Table 3) and DFS (Table 4). The Odds Ratio (OR) 95% CI and p-values are demonstrated in Table 3 for OS and Table 4 for DFS. Multivariate analysis using the cox-proportionality hazards model demonstrated TB as an independent prognostic factor for OS (OR 15.829; 95% CI 4.831–51.860; p-value <0.05) (Table 3) and DFS (OR 10.415; 95% CI 3.661–29.627; p-value<0.05) (Table 4) in our study cohort. The other parameters failed to reach significance on multivariate analysis.

**Table 3:** Univariate and multivariate survival analysis using Cox proportional hazards model for OS

**Parameters**	**Overall Survival (Univariate)**			**Overall Survival (Multivariate)**		
	**HR**	**95% CI**	**p value**	**HR**	**95% CI**	**p value**
**Depth of invasion**						
< 5 mm	*Reference*			*Reference*		
5-10 mm	1.63	0.795 - 3.342	0.183	1.161	0.559 - 2.413	0.689
> 10 mm	2.672	1.439 - 4.959	0.002*	1.609	0.783 - 3.305	0.195
**Worst pattern of invasion**						
WPOI 1, 2, 3, 4	*Reference*			*Reference*		
WPOI 5	1.65	1.028 - 2.647	0.038*	0.987	0.597 - 1.631	0.959
**Tumor budding **						
Low	*Reference*			*Reference*		
Intermediate	7.707	2.275 - 26.112	0.001*	6.9	2.019 - 23.584	0.002*
High	18.17	5.661 - 58.321	<0.001*	15.829	4.831 - 51.860	<0.001*
**Perineural invasion**						
Absent	*Reference*			*Reference*		
Present	1.762	1.118 - 2.779	0.015*	1.067	0.660 - 1.722	0.792
**Lymphovascular invasion**						
Absent	*Reference*			*Not done*		
Present	0.667	0.093 - 4.803	0.687			
**TNM staging**						
PT1, PT2, PT3	*Reference*			*Reference*		
PT4	1.831	1.170 - 2.868	0.008*	0.99	0.572 - 1.711	0.97
**Extranodal extension**						
Absent	*Reference*			*Reference*		
Present	0.651	0.323 - 1.313	0.231			

*p values: Significant. **OR: **Odds ratio, **CI: **Confidence interval.

**Table 4:** Univariate and multivariate survival analysis using Cox proportional hazards model for DFS

**Parameters**	**Disease Free Survival (Univariate)**			**Disease Free Survival (Multivariate)**		
	**HR**	**95% CI**	**p-value**	**HR**	**95% CI**	**p-value**
**Depth of invasion**	** **	** **	** **	** **	** **	** **
< 5 mm.	*Reference*			*Reference*		
5-10 mm.	1.483	0.726 - 3.030	0.279	1.051	0.508 - 2.174	0.894
> 10 mm.	2.47	1.330 - 4.585	0.004*	1.617	0.784 - 3.337	0.193
**Worst pattern of invasion**						
WPOI 1, 2, 3, 4	*Reference*			*Reference*		
WPOI 5	1.537	0.947 - 2.493	0.082*	1.008	0.604 - 1.681	0.976
**Tumor budding**						
Low	*Reference*			*Reference*		
Intermediate	5.75	1.876 - 17.626	0.002*	4.739	1.588 - 14.142	0.005*
High	13.697	4.758 - 39.425	<0.001*	10.415	3.661 - 29.627	<0.001*
**Perineural invasion**						
Absent	*Reference*			*Reference*		
Present	0.629	0.398 – 0.996	0.048*	1.004	0.616 – 1.637	0.986
**Lymphovascular invasion**						
Absent	*Reference*			*Not done*		
Present	0.048	0 – 69.232	0.414			
**TNM staging**						
PT1, PT2, PT3	*Reference*			*Reference*		
PT4	1.523	0.970 – 2.392	0.068*	0.839	0.484 - 1.453	0.531
**Extranodal extension**						
Absent	*Reference*			*Not done*		
Present	1.129	0.564 - 2.260	0.732			

*p values: Significant. **OR: **Odds ratio, **CI: **Confidence interval.

## DISCUSSION

In 1985, Gabbert et al. identified and described TB as a histopathological feature at the infiltrative tumor front in colorectal carcinoma (1). He named it tumor dedifferentiation attributing it to the cytoarchitectural alterations (1). Later the term ‘budding’ was proposed by Morodomi et al. and Hase et al. as these nests were observed to be budding out from larger tumor masses (13,14). TB is a microscopic feature that is defined as the tumor cells either separated singly or as tiny clusters of 5 or less than 5 cells at the invasive tumor front (1). It is reported that TB corresponds to the aggressive biological behavior of the tumor (1). It has been said that the tumor cells at the infiltrative tumor front undergo EMT, thereby promoting their activation, proliferation, and migration (1). 

TB has been studied extensively in colorectal carcinomas and has also been included in the College of American Pathologists (CAP) protocol for reporting colorectal carcinoma (15). It is considered as a major risk factor in predicting nodal metastasis in carcinomas arising from polyps as well as stage I and stage II adenocarcinomas of the colon (15). It is recently being studied extensively in other solid cancers, which include the oral cavity, nasopharynx, esophagus, urinary bladder, breast, lung, and pancreas (1). 

It was found that most of the published studies in head and neck squamous cell carcinoma have adopted a two tier grading system (high budding versus low budding), and the cutoff varied between 0 and 5 buds/field, 3 and 5 buds/field, and 5 and 10 buds/field (16). There is no common consensus to evaluate TB in OSCC. Hence, we extrapolated the ITBCC recommendations for evaluation of TB in colorectal carcinoma to the oral cavity (10). To the best of our knowledge, only three studies by Shimizu et al. (2018) (17), Xu et al. (2021) (9), and Tan and Taskin (2023) (18) have adopted the three-tiered system of grading TB proposed by the ITBCC consensus in OSCC, similar to our study. 

The present study showed interesting results. On univariate logistic analysis, TB, DOI (>10 mm), WPOI 5, and PNI were significantly associated with locoregional recurrence/distant metastasis. However, on multivariate logistic regression analysis, TB was found to be the most significant independent prognostic predictor for locoregional recurrence/distant metastasis. Univariate cox proportionality analysis showed TB, DOI (>10 mm), WPOI 5, pT4 stage, and PNI were associated with decreased overall survival and disease free survival in patients with OSCC. Nevertheless, multivariate cox proportionality analysis showed that TB was the only significant independent prognostic marker for decreased overall survival and disease free survival.

Shimizu et al. demonstrated that TB is a significant and independent prognostic marker for disease-free survival in patients with cT1-2 OSCC, underscoring its potential clinical value in risk stratification {HR = 2.18, 95% CI = 1.49–3.20, p<0.01} (17). However, the lack of a significant correlation between TB and WPOI suggests that these features may represent distinct aspects of tumor behavior, necessitating further investigation to clarify their roles in OSCC prognosis {p=0.14} (17). These results were found to be in concordance with our study results.

Xu et al. demonstrated that TB and WPOI are significantly associated with nodal metastasis in OSCC (9). High TB independently predicted a high risk of nodal metastasis {OR = 5.203, p < 0.001} (9). Univariate analysis showed that high TB correlated with worst overall survival, regional recurrence-free survival, and distant metastasis-free survival (9). However, in multivariate analysis, WPOI emerged as an independent prognostic factor for OS {HR = 1.994, p = 0.015}, while TB lost its statistical significance (9). These findings highlight the prognostic importance of WPOI over TB for OS in OSCC, though TB remains a strong predictor of nodal metastasis and other survival outcomes in univariate analysis (9).

Tan et al. investigated the prognostic significance of TB, PNI, and tumor cell nest size in overall survival. Univariate analysis revealed TB as a significant prognostic factor {HR = 4.786, 95% CI: 1.622–14.119, p < 0.001}, alongside with PNI and tumor cell nest size (18). However, in multivariate analysis, TB lost its significance, with PNI emerging as the only independent prognostic factor for survival (18). While TB is associated with overall survival in univariate analysis, PNI holds greater prognostic value as an independent marker (18). This underscores the importance of focusing on PNI in assessing survival outcomes (18).

Almangush et al. found that TB and DOI were significantly associated with mortality in tongue carcinoma (19). The authors used a two tiered grading system for the evaluation of TB (19). Univariate analysis confirmed that both TB and DOI were linked to disease-related death, and multivariate analysis retained TB, DOI, and WPOI as significant prognostic factors in tongue squamous cell carcinoma (19). A 2018 meta-analysis by Almangush et al. further supported these findings, showing that more than 5 tumor buds were associated with poor disease-free survival {HR = 1.83, 95% C; 1.34–2.50} and poor overall survival {HR = 1.88, 95% CI; 1.25-2.83} in OSCC (20). These studies emphasize the prognostic value of TB and DOI in predicting outcomes for tongue squamous cell carcinoma and OSCC (19,20). This evidence underscores the importance of these markers for risk stratification and highlights their potential utility in clinical decision-making (19,20).

In contrast to the studies done by Almangush et al, the current study used a three-tier system for evaluating TB intensity (low, intermediate, and high), following the ITBCC 2016 guidelines (10). This approach, supported by robust statistical evidence, offers clinically useful cutoffs for evaluating TB in OSCC. These findings emphasize the prognostic importance of TB and suggest that using a standardized approach, like the ITBCC 2016 guidelines, could enhance the clinical utility of TB as a predictor for patient outcomes in OSCC.

TB in OSCC is a current topic of increasing interest in cancer research, especially for its potential ability to predict patient outcomes. Our findings suggest that the disseminated tumor cells at the invasive front, known as TB, may play an important role in predicting survival and prognosis in patients with OSCC. This is consistent with previous research in several malignancies, which has identified TB as a microscopic prognosticator for predicting survival. The idea for a consistent, reliable technique for counting and scoring TB emphasizes the existing lack of a standardized methodology to evaluate this histological finding in OSCC. Achieving uniformity is imperative for facilitating the comparison of results across diverse studies and medical centers, as well as for the potential amalgamation of TB into standard histopathological reporting practices. By extrapolating the recommendations provided by the ITBCC, this research endeavor seeks to align with international standards for assessing TB. This is crucial for ensuring that the outcomes can be compared with research from other countries. As a result, we propose developing unified criteria for assessing tumor buds in OSCC, similar to colorectal cancer, in order to assure consistent global assessment.

The suggestion for broader, multi-center research at the conclusion indicates that, while our current work provides valuable insights, larger-scale research is required to validate and build on these findings. The emphasis on TB as a microscopic parameter for predicting the aggressiveness of OSCC suggests that understanding this histopathological parameter will have a significant impact on treatment choices and patient care for patients with OSCC in the near future. 

## CONCLUSION

In conclusion, our findings suggest that TB is a strong and an independent prognostic predictor in OSCC. While multiple histological parameters were significantly associated with poor outcomes in univariate analysis, TB emerged as the most powerful predictor in multivariate models. TB specifically predicted locoregional recurrence, distant metastasis, poor overall survival, and reduced disease-free survival. These findings highlight the significance of reporting. This could aid in classifying OSCC patients’ risk according to TB in conjunction with other independent, validated prognosticators like WPOI 5, DOI (Greater than 10 mm), PNI, LVI, and ENE, which could ultimately predict the prognosis as well as dictate the course of treatment.

## Funding

This study was not supported by any funding.

## Conflict of Interest

The authors declare that they have no conflict of interest.

## Ethical Approval

For this type of study formal consent is not required. This article does not contain any studies with human participants performed by any of the authors. This article does not contain any studies with animals performed by any of the authors. This article does not contain any studies with human participants or animals performed by any of the authors.

## Consent to Participate

For this type of study informed consent is not required. This study has obtained IRB approval from Malabar Cancer Center and the need for informed consent was waived. IRB number: 1616/IRB-SRC/13/MCC/10-03-2023/2)

## Consent for Publication

For this type of study consent for publication is not required.

## Availability of Data and Materials

A minimal dataset is available to interpret, replicate, and build upon the findings reported in the article.
